# Metabolic Tumor Volume from ^18^F-FDG PET/CT in Combination with Radiologic Measurements to Predict Long-Term Survival Following Transplantation for Colorectal Liver Metastases

**DOI:** 10.3390/cancers16010019

**Published:** 2023-12-19

**Authors:** Harald Grut, Pål-Dag Line, Trygve Syversveen, Svein Dueland

**Affiliations:** 1 Department of Radiology, Vestre Viken Hospital Trust, 3004 Drammen, Norway; 2 Institute of Clinical Medicine, University of Oslo, 0424 Oslo, Norway; 3 Department of Transplantation Medicine, Oslo University Hospital, 0424 Oslo, Norway; 4 Department of Radiology and Nuclear Medicine, Oslo University Hospital, 0424 Oslo, Norway

**Keywords:** ^18^F-FDG PET/CT, metabolic tumor volume, colorectal cancer, liver transplantation, liver metastases

## Abstract

**Simple Summary:**

Liver transplantation is emerging as a treatment option for patients with colorectal liver metastases. Due to the lack of liver donors, patient selection is vital. In this study, we report on the predictive value of combining metabolic tumor volume obtained from the pre-transplantation ^18^F-FDG PET/CT with the radiological measurements of tumor load (size, number, and tumor burden score) to select patients with the probability of long-term survival. Patients with low metabolic tumor volume have long disease-free survival, overall survival, and survival after relapse despite a high number of liver metastases and a high tumor burden score. This underlines the prognostic properties of metabolic active-tumor burden beyond conventional measurements.

**Abstract:**

The aim of the present study is to report on the ability of metabolic tumor volume (MTV) of liver metastases from pre-transplant ^18^F-FDG PET/CT in combination with conventional radiological measurements from CT scans to predict long-term disease-free survival (DFS), overall survival (OS), and survival after relapse (SAR) after liver transplantation for colorectal liver metastases. The total liver MTV was obtained from the ^18^F-FDG PET/CT, and the size of the largest metastasis and the total number of metastases were obtained from the CT. DFS, OS, and SAR for patients with a low and high MTV, in combination with a low and high size, number, and tumor burden score, were compared using the Kaplan–Meier method and log–rank test. Patients with a low number of metastases and low MTV had a significantly longer OS than those with a high MTV, with a median survival of 151 vs. 26 months (*p* = 0.010). Patients with a high number of metastases and low MTV had significantly longer DFS, OS, and SAR than patients with a high MTV (*p* = 0.034, 0.006, and 0.026). The tumor burden score of group/zone 3, in combination with a low MTV, had a significantly improved DFS, OS, and SAR compared to those with a high MTV (*p* = 0.034, <0.001, and 0.006). Patients with a low MTV of liver metastases had a long DFS, OS, and SAR despite a high number of liver metastases and a high tumor burden score.

## 1. Introduction

Most colorectal cancer patients with liver metastases have unresectable disease and receive palliative chemotherapy with a reported 5-year overall survival (OS) of only about 10% [1]. About 20–25% of patients with colorectal liver metastases (CRLMs) are evaluated to have resectable disease and undergo liver resection. Despite the curative intent of liver resection, most patients recur within 3 years, and the rate of relapse is related to the hepatic tumor load [2]. Most studies report a 5-year OS following resection of about 30–50% [3,4].

For selected patients diagnosed with hepatocellular carcinoma and metastases from low-grade neuroendocrine tumors, liver transplantation (LT) is a recognized treatment option [5,6,7]. The SEcondary CAncer (SECA) studies at the Oslo University Hospital show that an OS of about 80% can be achieved by LT in selected patients with CRLMs [8]. Based on this experience, multiple hospitals worldwide are currently conducting studies to explore LT for patients with CRLMs. However, common in the field of LT worldwide is the lack of liver donors. Thus, it is important to establish transplant criteria to select patients who have a long prospective post-transplant survival.

Computed tomography (CT) is, to date, still the workhorse in the pre-treatment work-up and follow-up of colorectal cancer patients. Liver magnetic resonance imaging (MRI) is increasingly used and recommended in the preoperative staging of potentially resectable CRLMs as it is reported to be more accurate for detection than CT [9,10]. The conventional way to evaluate the extent and dynamics of the disease on CT or MRI is to measure the size and number of metastases on repeated scans. Fluorine-18 fluorodeoxyglucose positron emission tomography in combination with CT (^18^F-FDG PET/CT) contributes functional/metabolic information in addition to anatomic information from the CT. This has been shown to improve the evaluation of several cancer types, including colorectal cancer, where ^18^F-FDG PET/CT is often used to evaluate the extent of the disease before a tentative metastasectomy [11,12,13]. The SECA study protocol includes an ^18^F-FDG PET/CT before a tentative LT to detect extrahepatic disease [8].

CRLMs visual on ^18^F-FDG PET/CT represent the active tumor tissue and the total metabolic tumor volume (MTV) that can be obtained from all patients. A high MTV as a biomarker of tumor burden is associated with poor prognosis compared to patients with a low MTV in several cancers, including patients with CRLMs [14,15,16].

The main objective of the present study was to evaluate the ability of MTV from ^18^F-FDG PET/CT, in combination with conventional measurements from CT, to predict 10-year survival following LT for CRLMs.

## 2. Materials and Methods

### 2.1. Patient Selection

In Norway (5.4 million inhabitants), transplantation surgery is centralized to the Oslo University Hospital. Hence, all patients who may be candidates for LT in general and for LT studies in Norway are referred at the Oslo University Hospital and evaluated at weekly multidisciplinary meetings consisting of a hepatobiliary/transplant surgeon, a radiologist, and an oncologist.

In total, 45 patients underwent LT for CRLMs in the SECA-1 (*n* = 23) and SECA-2 (*n* = 22) studies in the period from November 2006 to November 2018. The ^18^F-FDG PET/CT scan was part of the preoperative study protocol mainly to exclude extrahepatic disease [8]. Patients who fulfilled the inclusion criteria without extrahepatic malignant disease on ^18^F-FDG PET/CT and contrast-enhanced CT underwent an LT. All liver metastases from these ^18^F-FDG PET/CT scans were evaluated in the present study. All patients paused chemotherapy during the last 4–6 weeks before the ^18^F-FDG PET/CT scan. All inclusion/exclusion criteria, immunosuppression used, and follow-up regimen after LT were reported previously [8].

The SECA studies were approved by the Regional Ethics Committee, and all patients signed a written consent before inclusion. The studies are registered at clinicaltrials.gov (NCT01311453 for the SECA-1 study and NCT01479608 for the SECA-2 study).

### 2.2. Imaging Procedure and Image Assessments

The ^18^F-FDG PET/CT scans were performed on hybrid PET/CT scanners (Siemens Biograph 64/16 or GE Healthcare Discovery MI). All patients fasted for a minimum of six hours before injecting ^18^F-FDG. Serum glucose was measured, and the patients rested for about 60 min before image acquisition. A whole-body PET from the skull base to the upper thigh was performed. The PET was performed in combination with a non-contrast-enhanced low-dose CT for attenuation correction and anatomical information.

A Siemens Syngovia workstation (version VB10A, Erlangen, Germany) was used for a retrospective PET image assessment. MTV was obtained by placing a volume of interest over each metastasis using a 40% fixed threshold. MTV was registered if the maximum standardized uptake value in the metastasis was higher than the mean liver background uptake × 1.5 + standard deviation of the liver background × 2 [17]. The liver background was obtained by placing a 3 cm region of interest in the right liver lobe. The total MTV was calculated by adding the MTV from all metastases for each patient. The size and number of all liver metastases on the last CT before LT were measured and registered by a dedicated study radiologist.

### 2.3. Statistical Analysis

SPSS (IBM, version 27, Chicago, IL, USA) was used for statistical analyses. For the categories, including the number of metastases, the size of the largest metastasis, and MTV, the patients were divided into two groups (low or high) based on previously established cut-off values [18]. For the tumor burden score (TBS), the patients were placed in either group/zone 2 or 3 [19]. A high number of metastases was defined as ≥9; a large size was defined as a maximal tumor diameter of ≥5.5 cm, and a high MTV was defined as >70 cm^3^. OS was the time from LT until death or the end of follow-up (1st of February 2023). Disease-free survival (DFS) was defined as the time from LT until a visible relapse (local or metastases) on the CT/MRI/PET scan. Survival after relapse (SAR) was calculated as OS minus DFS in patients with recurrence. Survival estimates were made using the Kaplan–Meier (KM) method, and the groups were compared using the log–rank test. Synchronous liver metastases were defined as metastases occurring less than 12 months, with metachronous liver metastases occurring more than 12 months after the CRC diagnosis. A *p*-value less than 0.05 was considered statistically significant. The TBS^2^ = the maximum tumor diameter^2^ + number of metastases^2^ [19]. Respectively, a TBS of <3, 3–9, and >9 is classified as TBS zones 1, 2, and 3.

## 3. Results

### 3.1. Patient and Baseline Characteristics

The patient and baseline characteristics are given in Table 1. Out of the 45 included patients, 21 (47%) were women, and the median age for all included patients was 58 years (range 29–71).

### 3.2. Overall Survival Analysis

**Figure 1:**
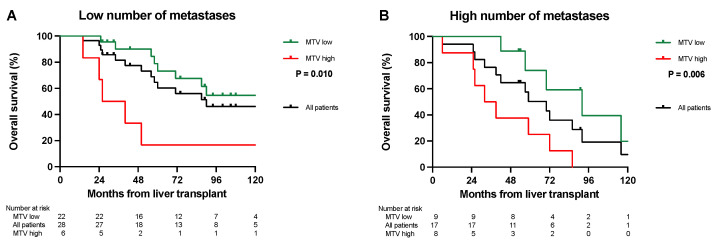
Overall survival. (**A**) Kaplan–Meier survival curve. Patients with a low number of metastases and a low MTV (green line) had significantly longer overall survival (*p* = 0.010) compared to those with a high MTV (red line). The black line shows the overall survival of all patients with a low number of metastases pooled (*n* = 28). (**B**) Kaplan–Meier survival curve. Patients with a high number of metastases and a low MTV (green line) had a significantly longer overall survival (*p* = 0.006) compared to patients with a high MTV (red line). The black line shows the overall survival for all patients with a high number of metastases pooled (*n* = 17).

In patients with a low number of metastases (*n* = 28) on CT, the patients with a low MTV (*n* = 22) had a median OS of 151 months, with an OS at 5 and 10 years of 79% and 55%. The six patients with a low number of metastases had a high MTV and a median OS of 26 months, with 17% OS at 5 and 10 years (Figure 1A, *p* = 0.010). In patients with a high number of metastases (*n* = 17), patients with a low MTV (*n* = 9) had a median survival of 91 months with OS at 5 and 10 years at 74% and 10%. The eight patients with a high MTV had a median OS of 32 months with OS at 5 and 10 years of 25% and 0% (Figure 1B, *p* = 0.006).

**Figure 2:**
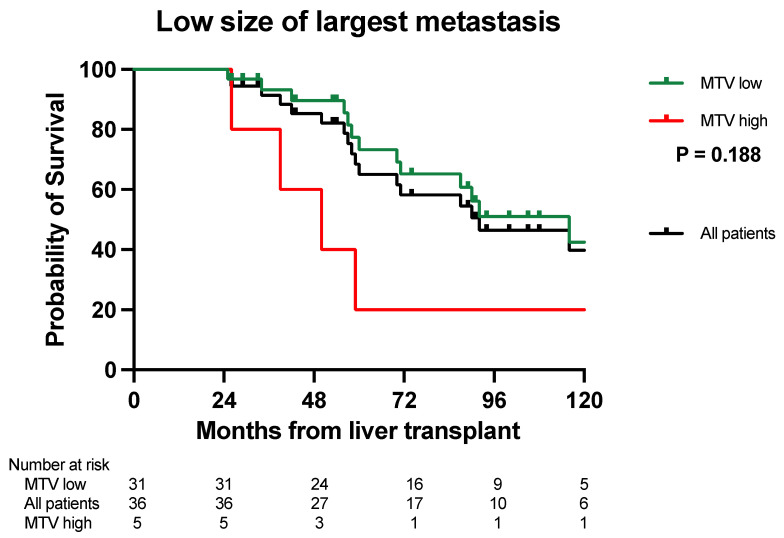
Kaplan–Meier survival curve. Patients with a low size of the largest metastasis and a low MTV (green line) had a longer overall survival (*p* = 0.188) than patients with a high MTV (red line). The black line shows the overall survival for all patients with a low number of metastases pooled (*n* = 36).

The median OS for patients with a small maximal diameter (*n* = 36) on CT and a low MTV (*n* = 31) was 116 months, with a 5- and 10-year survival of 77% and 43%. The five patients with a high MTV had a median OS of 50 months and 5- and 10-year survival of 20% (Figure 2, *p* = 0.188). The median survival of patients with a large size of liver metastases and a high MTV (*n* = 9) was 26 months. No patients had both a large size combined with a low MTV.

**Figure 3:**
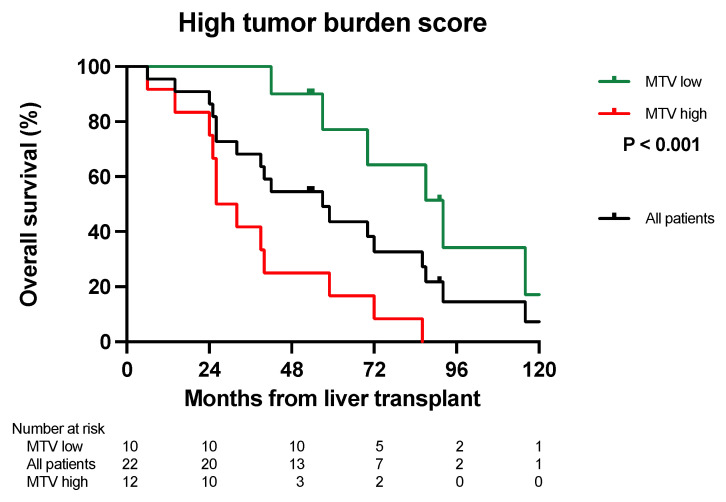
Kaplan–Meier survival curve. Patients with a high tumor burden score (group 3) and a low MTV (green line) had significantly longer overall survival (*p* < 0.001) compared to those with a high MTV (red line). The black line shows the overall survival for all patients with a high tumor burden score (group 3) pooled (*n* = 22).

The median survival for patients in TBS zone 2 and a low MTV (*n* = 20) was 151 months, with a 5- and 10-year OS of 78% and 58%. Only two patients were in the TBS zone 2 with a high MTV. One died 50 months post-LT, and the other patient is still alive, more than 13 years post-LT. The median survival of patients within the TBS zone 3 (*n* = 22) and a low MTV (*n* = 10) was 92 months, with a 5- and 10-year survival at 77% and 17%. The median survival of patients in the TBS zone 3 and a high MTV was 26 months, with 5- and 10-year survival at 17% and 0% (Figure 3, *p* < 0.001). One patient in TBS zone 1 was not included. This patient is alive 33 months after LT without recurrence.

### 3.3. Disease-Free Survival Analysis

An overview of DFS (%) at 1, 3, and 5 years is given in Table 2. The median DFS in patients with a low number of metastases and a low MTV (*n* = 22) was 22 months compared with 6 months in six patients with a high MTV (*p* = 0.010). Patients with a high number of metastases and a low MTV (*n* = 9) had a median DFS of 11 months versus 3 months in eight patients with a high MTV (*p* = 0.034). Patients with a low maximal tumor diameter and a low MTV (*n* = 31) had a median DFS of 19 months compared with 3 months in four patients with a high MTV (*p* = 0.002). No patients had a combination of large and low MTV. The median DFS for patients in TBS score zone 2 and a low MTV (*n* = 21) was 23 months and 3 months in the two patients with a high MTV (*p* = 0.086). Patients in TBS zone 3 and a low MTV (*n* = 10) and a median DFS of 11 months compared to 6 months for the six patients with high MTV (*p* = 0.034).

### 3.4. Survival after Relapse Analysis

**Figure 4:**
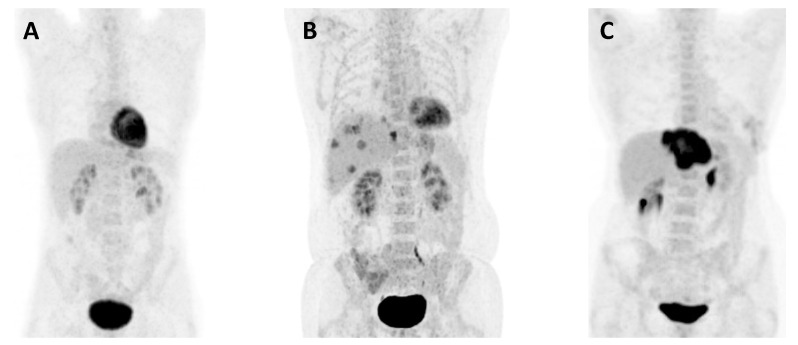
Image examples. (**A**) This patient had a low MTV (0 cm^3^) and 40 liver metastases on the CT, and the largest metastasis was 38 mm with a TBS of 40.2. The patient is still alive more than 12 years after LT. (**B**) This patient had a low MTV (46.35 cm^3^), 25 liver metastases on CT, the largest metastasis was 35 mm, and a TBS of 25.2. The patient is still alive more than 5 years after LT. (**C**) This patient had a high MTV (146.50 cm^3^), 7 liver metastases on the CT, the largest metastasis was 105 mm, and a TBS of 12.6. The patient died only 2 years after LT.

An overview of SAR (%) at 1, 3, and 5 years is given in Table 3. The median SAR in patients with a low number of metastases and a low MTV (*n* = 15) was 66 months compared with 20 months in six patients with a high MTV (*p* = 0.144). Patients with a high number of metastases and a low MTV (*n* = 7) had a median SAR of 89 months versus 24 months in eight patients with a high MTV (*p* = 0.026). Patients with a low maximal tumor size and a low MTV (*n* = 22) had a median SAR of 82 months compared with 36 months in four patients with a high MTV (*p* = 0.749). The median SAR in the nine patients with a large size and a high MTV was 20 months. No patients had a combination of large size and low MTV. The median SAR for patients in TBS zone 2 and a low MTV (*n* = 14) was 66 months, and 37 months in patients with a high MTV (*n* = 2). Patients in TBS zone 3 and a low MTV (*n* = 8) had a median DFS of 11 months compared to 6 months in the 12 patients with a high MTV (*p* = 0.006). Figure 4 shows image examples of three SECA patients.

## 4. Discussion

Deceased donor liver grafts are a limited source. The selection of patients is, therefore, of crucial importance to avoid the futile use of liver grafts. For most indications, a prospective survival rate of 5 years of about 70–75% is required to offer patients a liver transplant. LT in patients with CRLMs has been a controversial indication for this procedure. However, through careful selection, a 5-year OS of 70–75% might be obtained [8,18]. We have previously shown that several prognostic factors, including PET liver MTV with a cut-off of 70 cm^3^, a number of liver lesions larger than nine, the size of the largest liver lesion being >5.5 cm, and TBS zone 2 vs. 3, can stratify patients into groups with a good and poor 5-year OS [18,20].

In this report, we combined the tumor load from CT scans and PET-MTV values to determine the “biologically active tumor volume”. This is clinically relevant if it can be utilized to select patients with inferior survival but with a good prognostic score based on their radiological tumor load. Similarly, this approach could be utilized to select patients with long OS from patients with a higher tumor load and perceived bad prognosis. Our results suggest that by combining CT scans and PET liver MTV values, survival rates comparable to conventional LT indications might be obtained with a 5-year OS of 74–79% (Figure 1A,B and Figure 2). Furthermore, this combination might also identify patients with “bad tumor biology” and inferior 5-year OS after LT that should not be considered for transplantation (Figure 1A,B and Figure 2). MTV values combined with CT scans also separated patients into different DFS and SAR groups (Table 2 and Table 3), and the long SAR seen in patients with a low MTV suggests that this parameter is closely related to the “tumor biology” of the disease.

MTV is usually easy to obtain and may be included in the clinical routine for prognostic purposes in conjunction with conventional measurements. To reflect the biologically active tumor burden at LT, the ^18^F-FDG PET/CT should be performed as close to LT as practically possible [20]. We previously showed that ^18^F-FDG PET/CT is important to detect extrahepatic disease in addition to CT prior to LT [21]. Extrahepatic disease is a contraindication to LT, and in long LT waiting times, a repeated PET scan is advised after 3 months to avoid the inclusion of patients with new extrahepatic disease undetected by conventional contrast-enhanced CT [20].

All SECA patients received chemotherapy prior to LT with a 4–6 week pause before the ^18^F-FDG PET/CT scan. The MTV from this scan represents the remaining metabolically active liver metastases after chemotherapy and may be a surrogate marker of the response to chemotherapy. Patients with high levels of MTV probably had liver metastases with a more aggressive tumor biology compared to those with low levels of MTV. It is plausible that patients with aggressive tumor biology are prone to developing extrahepatic metastases, and this is more relevant for the prediction of survival than the absolute number of lesions. In line with our results, other studies have shown that ^18^F-FDG PET is predictive of response and survival following neoadjuvant chemotherapy in locally advanced rectal cancer and the response of colorectal cancer metastases after chemotherapy [22]. Furthermore, we showed that PET-liver MTV values are related to OS after the resection of pulmonary metastases after LT, suggesting that PET-liver MTV values might represent tumor biology [23].

Unfortunately, it is not possible to detect micrometastases despite the use of several image modalities like CT, MRI, and PET. CRLMs in most unresectable patients is a systemic disease, and most patients in the SECA studies relapsed, with about 2/3 developing indolent pulmonary metastases [8,24,25]. However, several reports indicate that most patients with pulmonary relapse can be treated with a curative intent, whereas patients with extrapulmonary recurrence have short survival [8,24]. Thus, in this setting, the site of recurrence is more important than disease-free survival (DFS). Because of the poor correlation between DFS and OS, DFS is of limited value as a parameter of treatment efficacy in LT for CRLMs. This is different from patients undergoing LT for HCC, where few patients recur, but those who do have short survival [26,27]. Several studies have shown that PET-derived MTV may predict recurrence and survival for these patients [28,29]. Furthermore, MTV from ^18^F-FDG PET/CT is also prognostic in patients undergoing selective internal radiation therapy with yttrium-90 for unresectable CRLMs, as well as in patients undergoing liver resection for CRLMs [14,30,31].

Our study has some limitations. This study was retrospective, and the number of patients was low (*n* = 45). Despite the relatively low number of patients, the present study showed significant results. Also, the SECA study is the largest controlled trial on LT for CRLMs worldwide, with a very long observation time. The PET scans in the SECA studies followed a clinical standard protocol without motion correction. Consequently, the smearing of uptake may have affected the measurements. There is no consensus on which threshold to use in the segmentation of tumors. As for the initial SECA-1 patients, a 40% fixed threshold was used for all patients in this study to be consistent. The use of other thresholds could have affected the uptake values, but it is unlikely that this would significantly alter the stratification of patients into an MTV low and an MTV high group.

## 5. Conclusions

Patients with a low MTV have a long DFS, OS, and SAR after LT despite a high number of lesions and a high tumor burden score. Patients with eight lesions or less or size of the largest metastasis less than 5.5 cm had inferior OS after LT if they had high MTV values. ^18^F-FDG PET/CT combined with conventional measurements can select colorectal cancer patients with liver-only metastases that can obtain a 5-year OS comparable to other indications for LT.

## Figures and Tables

**Table 1:** Patient and baseline characteristics.

Patients, *n*	45
Age at liver transplantation (LT), years ^+^	58 (29–71)
Gender, *n* (%)	
Male	24 (53)
Female	21 (47)
Primary tumor, *n* (%)	
Colon	27 (60)
Rectum	18 (40)
T Stage, *n* (%)	
0	3 (6)
1	1 (2)
2	5 (10)
3	35 (78)
4	1 (2)
N Stage, *n* (%)	
0	21 (47)
1	16 (36)
2	8 (18)
Progressive disease at LT, *n* (%) *	
Yes	16 (36)
No	29 (64)
Metabolic tumor volume (MTV), cm^3 +^	21.3 (0–874.12)
Carcinoembryonic antigen (CEA), µg/L ^+^	5 (1–2002)
Number of metastases, *n* ^+^	7 (1–53)
Largest metastasis (mm) ^+^	30 (3–130)

^+^ median (range); * Increased size of liver metastases and/or increased carcinoembryonic antigen (CEA).

**Table 2:** Results. Disease-free survival.

Group		Disease-Free Survival						
		MTV Low			MTV High			
		1 Year	3 Years	5 Years	1 Year	3 Years	5 Years	*p*-Value
Number of metastases	Low (≤8)	59	41%	34%	17%	0%	0%	0.010
	High (>8)	44%	33%	22%	25%	0%	0%	0.034
Size of the largest metastasis	Low (<5.5 cm)	55%	39%	29%	25%	0%	0%	0.002
	High (>5.5 cm)	*n* = 0			22%	0%	0%	
Tumor burden score	Group 2	62%	43%	36%	50%	0%	0%	0.086
	Group 3	40%	30%	20%	17%	0%	0%	0.034

**Table 3:** Results. Survival after relapse.

Group		Survival after Relapse				
		MTV Low		MTV High		
		5 Years	10 Years	5 Years	10 Years	*p*-Value
Number of metastases	Low (≤8)	54%	39%	17%	0%	0.144
	High (>8)	57%	19%	13%	0%	0.026
Size of the largest metastasis	Low (<5.5 cm)	60%	31%	25%	0%	0.749
	High (>5.5 cm)	*n* = 0		11%	0%	
Tumor burden score	Group 2	50%	42%	*n* = 2		0.604
	Group 3	75%	16%	8%	0%	0.006

## Data Availability

The data are not available due to legal and privacy restrictions.

## References

[1] Dueland S., Guren T.K., Hagness M., Glimelius B., Line P.D., Pfeiffer P., Foss A., Tveit K.M. (2015). Chemotherapy or liver transplantation for nonresectable liver metastases from colorectal cancer?. Ann. Surg..

[2] Allard M.A., Adam R., Giuliante F., Lapointe R., Hubert C., Ijzermans J.N.M., Mirza D.F., Elias D., Laurent C., Gruenberger T. (2017). Long-term outcomes of patients with 10 or more colorectal liver metastases. Br. J. Cancer.

[3] Nordlinger B., Sorbye H., Glimelius B., Poston G.J., Schlag P.M., Rougier P., Bechstein W.O., Primrose J.N., Walpole E.T., Finch-Jones M. (2013). Perioperative FOLFOX4 chemotherapy and surgery versus surgery alone for resectable liver metastases from colorectal cancer (EORTC 40983): Long-term results of a randomised, controlled, phase 3 trial. Lancet Oncol..

[4] Quan D., Gallinger S., Nhan C., Auer R.A., Biagi J.J., Fletcher G.G., Law C.H., Moulton C.A., Ruo L., Wei A.C. (2012). The role of liver resection for colorectal cancer metastases in an era of multimodality treatment: A systematic review. Surgery.

[5] Mazzaferro V., Llovet J.M., Miceli R., Bhoori S., Schiavo M., Mariani L., Camerini T., Roayaie S., Schwartz M.E., Grazi G.L. (2009). Predicting survival after liver transplantation in patients with hepatocellular carcinoma beyond the Milan criteria: A retrospective, exploratory analysis. Lancet Oncol..

[6] Le Treut Y.P., Gregoire E., Klempnauer J., Belghiti J., Jouve E., Lerut J., Castaing D., Soubrane O., Boillot O., Mantion G. (2013). Liver transplantation for neuroendocrine tumors in Europe-results and trends in patient selection: A 213-case European liver transplant registry study. Ann. Surg..

[7] Mazzaferro V., Bhoori S., Sposito C., Bongini M., Langer M., Miceli R., Mariani L. (2011). Milan criteria in liver transplantation for hepatocellular carcinoma: An evidence-based analysis of 15 years of experience. Liver Transpl..

[8] Dueland S., Syversveen T., Solheim J.M., Solberg S., Grut H., Bjornbeth B.A., Hagness M., Line P.D. (2019). Survival Following Liver Transplantation for Patients with Nonresectable Liver-only Colorectal Metastases. Ann. Surg..

[9] Cervantes A., Adam R., Roselló S., Arnold D., Normanno N., Taïeb J., Seligmann J., De Baere T., Osterlund P., Yoshino T. (2023). Metastatic colorectal cancer: ESMO Clinical Practice Guideline for diagnosis, treatment and follow-up. Ann. Oncol..

[10] Tsili A.C., Alexiou G., Naka C., Argyropoulou M.I. (2021). Imaging of colorectal cancer liver metastases using contrast-enhanced US, multidetector CT, MRI, and FDG PET/CT: A meta-analysis. Acta Radiol..

[11] Briggs R.H., Chowdhury F.U., Lodge J.P., Scarsbrook A.F. (2011). Clinical impact of FDG PET-CT in patients with potentially operable metastatic colorectal cancer. Clin. Radiol..

[12] Lin M., Wong K., Ng W.L., Shon I.H., Morgan M. (2011). Positron emission tomography and colorectal cancer. Crit. Rev. Oncol. Hematol..

[13] (2023). National Comprehensive Cancer Network. https://www.nccn.org/professionals/physician_gls/pdf/colon.pdf.

[14] Kim Y.I., Lee H.S., Choi J.Y. (2021). Prognostic Significance of Pretreatment ^18^F-FDG PET/CT Volumetric Parameters in Patients with Colorectal Liver Metastasis: A Systematic Review and Meta-analysis. Clin. Nucl. Med..

[15] Makino T., Yamasaki M., Tanaka K., Masuike Y., Tatsumi M., Motoori M., Kimura Y., Hatazawa J., Mori M., Doki Y. (2019). Metabolic Tumor Volume Change Predicts Long-term Survival and Histological Response to Preoperative Chemotherapy in Locally Advanced Esophageal Cancer. Ann. Surg..

[16] Pellegrino S., Fonti R., Mazziotti E., Piccin L., Mozzillo E., Damiano V., Matano E., De Placido S., Del Vecchio S. (2019). Total metabolic tumor volume by ^18^F-FDG PET/CT for the prediction of outcome in patients with non-small cell lung cancer. Ann. Nucl. Med..

[17] Wahl R.L., Jacene H., Kasamon Y., Lodge M.A. (2009). From RECIST to PERCIST: Evolving Considerations for PET response criteria in solid tumors. J. Nucl. Med..

[18] Dueland S., Smedman T.M., Syversveen T., Grut H., Hagness M., Line P.D. (2023). Long-Term Survival, Prognostic Factors, and Selection of Patients with Colorectal Cancer for Liver Transplant: A Nonrandomized Controlled Trial. JAMA Surg.

[19] Sasaki K., Morioka D., Conci S., Margonis G.A., Sawada Y., Ruzzenente A., Kumamoto T., Iacono C., Andreatos N., Guglielmi A. (2018). The Tumor Burden Score: A New “Metro-ticket” Prognostic Tool for Colorectal Liver Metastases Based on Tumor Size and Number of Tumors. Ann. Surg..

[20] Grut H., Line P.D., Syversveen T., Dueland S. (2022). Metabolic tumor volume predicts long-term survival after transplantation for unresectable colorectal liver metastases: 15 years of experience from the SECA study. Ann. Nucl. Med..

[21] Grut H., Revheim M.E., Line P.D., Dueland S. (2018). Importance of ^18^F-FDG PET/CT to select patients with nonresectable colorectal liver metastases for liver transplantation. Nucl. Med. Commun..

[22] Jadvar H., Colletti P.M., Delgado-Bolton R., Esposito G., Krause B.J., Iagaru A.H., Nadel H., Quinn D.I., Rohren E., Subramaniam R.M. (2017). Appropriate Use Criteria for (18)F-FDG PET/CT in Restaging and Treatment Response Assessment of Malignant Disease. J. Nucl. Med..

[23] Dueland S., Smedman T.M., Grut H., Syversveen T., Jørgensen L.H., Line P.D. (2022). PET-Uptake in Liver Metastases as Method to Predict Tumor Biological Behavior in Patients Transplanted for Colorectal Liver Metastases Developing Lung Recurrence. Cancers.

[24] Hagness M., Foss A., Egge T.S., Dueland S. (2014). Patterns of recurrence after liver transplantation for nonresectable liver metastases from colorectal cancer. Ann. Surg. Oncol..

[25] Grut H., Solberg S., Seierstad T., Revheim M.E., Egge T.S., Larsen S.G., Line P.D., Dueland S. (2017). Growth rates of pulmonary metastases after liver transplantation for unresectable colorectal liver metastases. Br. J. Surg..

[26] Chagas A.L., Felga G.E.G., Diniz M.A., Silva R.F., Mattos A.A., Silva R.C.M.A., Boin I.F.S.F., Garcia J.H.P., Lima A.S., Coelho J.C.U. (2019). Hepatocellular carcinoma recurrence after liver transplantation in a Brazilian multicenter study: Clinical profile and prognostic factors of survival. Eur. J. Gastroenterol. Hepatol..

[27] Foerster F., Hoppe-Lotichius M., Vollmar J., Marquardt J.U., Weinmann A., Wörns M.A., Otto G., Zimmermann T., Galle P.R. (2019). Long-term observation of hepatocellular carcinoma recurrence after liver transplantation at a European transplantation centre. United Eur. Gastroenterol. J..

[28] Ding E., Lu D., Wei L., Feng X., Shen J., Xu W. (2020). Predicting tumor recurrence using metabolic indices of (18)F-FDG PET/CT prior to orthotopic liver transplantationfor hepatocellular carcinoma. Oncol. Lett..

[29] Miao W., Nie P., Yang G., Wang Y., Yan L., Zhao Y., Yu T., Yu M., Wu F., Rao W. (2021). An FDG PET/CT metabolic parameter-based nomogram for predicting the early recurrence of hepatocellular carcinoma after liver transplantation. Eur. J. Nucl. Med. Mol. Imaging.

[30] Gulec S.A., Suthar R.R., Barot T.C., Pennington K. (2011). The prognostic value of functional tumor volume and total lesion glycolysis in patients with colorectal cancer liver metastases undergoing 90Y selective internal radiation therapy plus chemotherapy. Eur. J. Nucl. Med. Mol. Imaging.

[31] Soydal C., Kucuk O.N., Gecim E.I., Bilgic S., Elhan A.H. (2013). The prognostic value of quantitative parameters of ^18^F-FDG PET/CT in the evaluation of response to internal radiation therapy with yttrium-90 in patients with liver metastases of colorectal cancer. Nucl. Med. Commun..

